# Epidemiology and seasonality of respiratory viral infections in hospitalized children in Kuala Lumpur, Malaysia: a retrospective study of 27 years

**DOI:** 10.1186/1471-2431-12-32

**Published:** 2012-03-20

**Authors:** Chee-Sieng Khor, I-Ching Sam, Poh-Sim Hooi, Kia-Fatt Quek, Yoke-Fun Chan

**Affiliations:** 1 Tropical Infectious Diseases Research and Education Centre, Department of Medical Microbiology, Faculty of Medicine, University Malaya, Kuala Lumpur, Malaysia; 2 Diagnostic Virology Laboratory, University Malaya Medical Centre, Kuala Lumpur, Malaysia; 3 School of Medicine & Health Sciences, Monash University Malaysia, Bandar Sunway, Petaling Jaya, Selangor Darul Ehsan, Malaysia

## Abstract

**Background:**

Viral respiratory tract infections (RTI) are relatively understudied in Southeast Asian tropical countries. In temperate countries, seasonal activity of respiratory viruses has been reported, particularly in association with temperature, while inconsistent correlation of respiratory viral activity with humidity and rain is found in tropical countries. A retrospective study was performed from 1982-2008 to investigate the viral etiology of children (≤ 5 years old) admitted with RTI in a tertiary hospital in Kuala Lumpur, Malaysia.

**Methods:**

A total of 10269 respiratory samples from all children ≤ 5 years old received at the hospital's diagnostic virology laboratory between 1982-2008 were included in the study. Immunofluorescence staining (for respiratory syncytial virus (RSV), influenza A and B, parainfluenza types 1-3, and adenovirus) and virus isolation were performed. The yearly hospitalization rates and annual patterns of laboratory-confirmed viral RTIs were determined. Univariate ANOVA was used to analyse the demographic parameters of cases. Multiple regression and Spearman's rank correlation were used to analyse the correlation between RSV cases and meteorological parameters.

**Results:**

A total of 2708 cases were laboratory-confirmed using immunofluorescence assays and viral cultures, with the most commonly detected being RSV (1913, 70.6%), parainfluenza viruses (357, 13.2%), influenza viruses (297, 11.0%), and adenovirus (141, 5.2%). Children infected with RSV were significantly younger, and children infected with influenza viruses were significantly older. The four main viruses caused disease throughout the year, with a seasonal peak observed for RSV in September-December. Monthly RSV cases were directly correlated with rain days, and inversely correlated with relative humidity and temperature.

**Conclusion:**

Viral RTIs, particularly due to RSV, are commonly detected in respiratory samples from hospitalized children in Kuala Lumpur, Malaysia. As in temperate countries, RSV infection in tropical Malaysia also caused seasonal yearly epidemics, and this has implications for prophylaxis and vaccination programmes.

## Background

Acute respiratory tract infection (RTI) is a major cause of morbidity and mortality worldwide, particularly in children [1,2]. An estimated 1.9 million children die from acute RTI every year, with 70% of the mortality occurring in Africa and Southeast Asia [2]. Most respiratory tract infections are caused by viruses [3].

Respiratory viruses are generally transmitted through inhalation or direct contact with respiratory aerosols or secretions. Transmission is often associated with geographic and climatic factors. Lower temperatures, lower ultraviolet B radiance, and higher humidity prolong the survival rate of respiratory viruses in the environment [4,5]. In temperate countries, seasonal activity of respiratory viruses has been reported, particularly in association with temperature [4]. In tropical countries, correlation of respiratory viral activity with climatic factors are not so well defined, which may suggest that more complex interactions are involved [6].

Relatively few studies on viral RTIs have been conducted in Southeast Asian countries like Malaysia [7-9], despite reports from the Ministry of Health that RTI is one of the main causes of hospitalization in Malaysia [10]. The epidemiology of respiratory viruses needs to be established to increase the effectiveness of any planned vaccination and prophylaxis programmes. The lack of up-to-date basic epidemiological data on viral RTIs in Malaysian children needs to be addressed. In this study, we describe etiological agents, demographic details of patients, and seasonality (including association with meteorological factors) due to viral RTIs in a teaching hospital in Kuala Lumpur, over the last 27 years.

## Results

Over a 27-year period (1982-2008), 10269 samples from children ≤ 5 years were sent for respiratory virus detection, of which 2708 (26.4%) were positive for the common respiratory viruses. The numbers of samples received at the laboratory have been increasing in recent years, from an annual mean of 319 between 1982-1999 to an annual mean of 503 between 2000-2008, as the number of admissions has increased. The mean annual positive rate has been reasonably stable at 25.4% (standard deviation, 8.1%; range, 8.9-38.1%). The viruses detected were RSV (1913, 70.6%), parainfluenza viruses 1-3 (357, 13.2%), influenza A and B viruses (297, 11.0%), and adenovirus (141, 5.2%). The 310 typeable parainfluenza virus cases consisted of 93 (30.0%) parainfluenza-1 virus (PIV-1), 23 (7.4%) parainfluenza-2 (PIV-2) virus and 194 (62.6%) parainfluenza-3 (PIV-3) virus, while a further 47 cases could not be typed by immunofluorescence (IF). Influenza cases consisted of 233 (78.1%) influenza A and 64 (21.9%) influenza B. Co-infections were only detected in 26 cases, with co-infection of RSV and influenza A (n = 6), RSV and PIV-1 (n = 2), RSV and PIV-3 (n = 7), RSV and adenovirus (n = 3), influenza A and PIV-3 (n = 2), influenza A and adenovirus (n = 1), influenza B and PIV-1 (n = 1), influenza B and adenovirus (n = 1), PIV-3 and adenovirus (n = 1), RSV and influenza A and PIV-1 (n = 1). Among 792 samples with positive viral isolation, 442 samples were also positive by IF, giving an overall IF sensitivity of 55.8% (with viral isolation as a gold standard).

### Epidemiological data

The demographic data of these children are summarized in Table 1. The mean age of the study population was 1.14 ± 1.06 years old, and 76.2% of the positive cases were seen in children ≤ 1 year old. RSV was by far the commonest identified respiratory virus in children ≤ 6 months, accounting for 81.3% of the positive samples in this age group, but this declined to 56.7% in those aged 1-5 years, respectively. Correspondingly, the relative importance of influenza viruses and adenovirus increased with age in the ≤ 6 months to the 1-5 years age groups, from 5.5% to 20.2%, and 2.6% to 8.8%, respectively. The mean ages of children infected by each of the common viruses ranged from 0.96 to 1.51 years, and were significantly different by one-way ANOVA testing (F = 24.632, df = 4, p < 0.05). Post-hoc testing showed that children infected with RSV were significantly younger (mean difference = 0.178, SE = 0.024, p < 0.05), while children infected with influenza viruses were significantly older than the study population (mean difference = 0.372, SE = 0.065, p < 0.05).

**Table 1:** Distribution of respiratory viruses in children ≤ 5 years according to age group, gender and ethnicity

		Age group, n (%)			Total	Mean age ± SD (yrs)	Male/female ratio**	Ethnicity, n (%) **			
						
		≤ 6 months	6-12 months	1-5 yrs				Malay	Chinese	Indian	Others
Samples (% of total)	**Samples received**	3319 (32.3)	4241 (41.3)	2709 (26.4)	10269	1.14 ± 1.06	1.5	6337 (62.0)	2081 (20.4)	1596 (15.6)	199 (1.9)
	**Positive samples**	906 (33.5)	1157 (42.7)	645 (23.8)	2708	1.06 ± 1.00	1.49	1640 (61.5)	548 (20.6)	426 (16.0)	52 (2.0)

Respiratory virus identified (% of total positive samples within age/ethnic group)	**RSV**	737 (81.3)	810 (70.0)	366 (56.7)	1913 (70.6)	0.96 ± 0.92*	1.51	1137 (69.3)	407 (74.3)	326 (76.5)	33 (63.5)
	**Parainfluenza**	95 (10.5)	170 (14.7)	92 (14.3)	357 (13.2)	1.10 ± 0.92	1.6	211 (12.9)	83 (15.1)	48 (11.3)	7 (13.5)
	Parainfluenza 1	24 (2.6)	44 (3.8)	25 (3.9)	93 (3.4)	1.19 ± 1.00	1.51	60 (3.7)	17 (3.1)	15 (3.5)	1 (1.9)
	Parainfluenza 2	2 (0.2)	15 (1.3)	6 (0.9)	23 (0.8)	0.84 ± 0.86	1.75	16 (1.0)	2 (0.4)	1 (0.2)	1 (1.9)
	Parainfluenza 3	50 (5.5)	90 (7.8)	54 (8.4)	194 (7.2)	1.11 ± 0.90	1.49	112 (6.8)	49 (8.9)	25 (5.9)	3 (5.8)
	**Influenza**	50 (5.5)	117 (10.1)	130 (20.2)	297 (11.0)	1.51 ± 1.10*	1.2	192 (11.7)	44 (8.0)	40 (9.4)	8 (15.4)
	Influenza A	42 (4.6)	92 (8.0)	99 (15.3)	233 (8.6)	1.50 ± 1.10*	1.15	149 (9.1)	37 (6.8)	30 (7.0)	7 (13.5)
	Influenza B	8 (0.9)	25 (2.2)	31 (4.8)	64 (2.4)	1.56 ± 1.09*	1.42	43 (2.6)	7 (1.3)	10 (2.3)	1 (1.9)
	**Adenovirus**	24 (2.6)	60 (5.2)	57 (8.8)	141 (5.2)	1.37 ± 1.04	1.75	100 (6.1)	14 (2.6)	12 (2.8)	4 (7.7)

SD, standard deviation

* p < 0.05

** Of the 10269 total number of samples received, 85 and 56 were missing gender and ethnicity data, respectively.

Amongst the positive cases, 59.8% were male and 41.2% were female. Overall, the most commonly infected ethnic group were Malays (61.5%), followed by Chinese (20.6%), and Indians (16.0%), reflecting the ethnicities of the patients sampled. There were no significant differences in terms of gender and ethnicity between patients infected with different viruses.

### Seasonal activity of respiratory viruses

**Figure 1:**
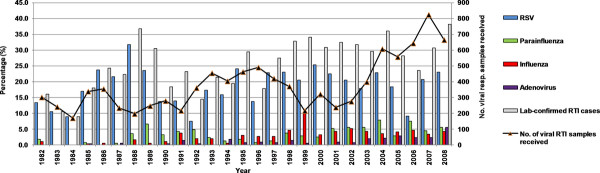
**Proportions of respiratory viruses detected between 1982 and 2008**. The detection of each respiratory virus as a proportion of the total number of positive respiratory virus samples is shown.

**Figure 2:**
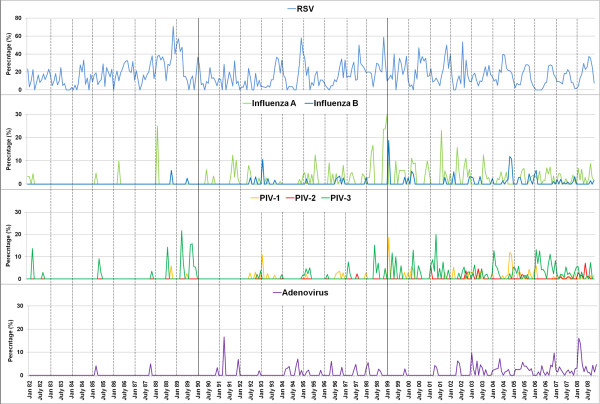
**Monthly activity pattern of respiratory viruses between 1982 and 2008**. The detection of respiratory viruses in each month was plotted over 27 years demonstrating the seasonal trends over the years. The percentage rates of detection of each virus (divided by the total number of samples positive for any respiratory virus) are shown.

**Figure 3:**
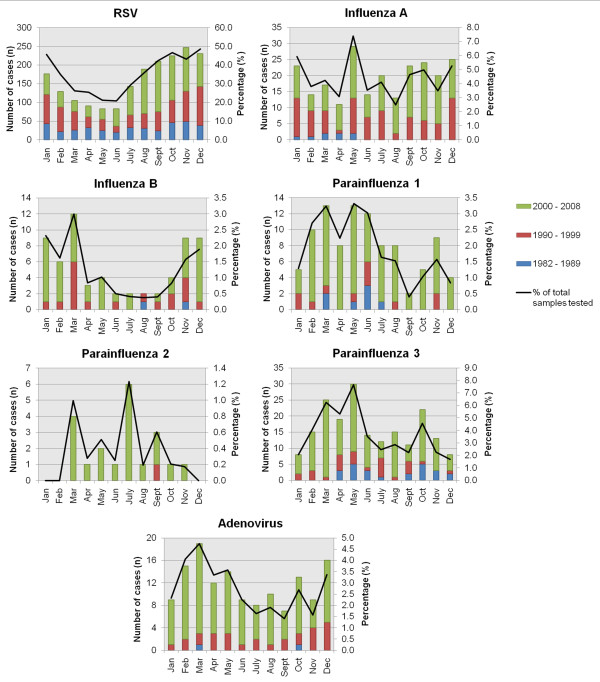
**Within-year seasonality patterns of respiratory viruses**. Cases over 27 years were aggregated according to the month the specimen was received. The figure includes percentage rates of detection of each virus when divided by the total number of samples tested. This shows that the observed monthly trends were not affected by the total number of samples received.

Due to the relatively small numbers of laboratory-confirmed cases for all other respiratory viruses other than RSV over the study period, particularly in the early years, it was hard to discern multi-year cycles of individual viruses (Figure 1, Figure 2). In the last decade, most viruses were detected every year, except for PIV2. To obtain a clearer picture of seasonality within a year, the cases due to each virus were also aggregated into months (Figure 3). Disease activity due to the main respiratory viruses was present throughout the year, with yearly peaks of activity. RSV showed the most pronounced seasonality, with peak activity at the year-end (September-December), and lowest activity in mid-year (April-June). PIV-1 and PIV-3 virus activity was mainly seen in March-May. The number of PIV-2 cases was too small to detect any seasonality. Influenza A was seen throughout the year, with peak activity in May, while there was more obvious increased influenza B virus activity between November-March. Adenovirus activity was present all year-round, with a peak in February-March.

### Climatic factors

Since RSV activity showed the clearest seasonal trend, and had the highest number of cases, the association of RSV with climatic factors was further analyzed (Table 2). Spearman's rank correlation and multiple regression showed direct correlation of monthly RSV cases with rain days and inverse correlation with temperature. Additionally, regression analysis identified a further significant inverse correlation of RSV cases with relative humidity. Multiple regression showed that a monthly increase of one rain day was associated with a 0.469 increase in monthly RSV cases. However, increases of 1% in relative humidity and 1°C in temperature were associated with 1.070 and 2.426 decreases in RSV cases, respectively. A total of 14.3% of explained variance (R^2 ^= 0.143) in the number of monthly RSV cases could be attributed to these three factors. Other climatic factors were not significantly associated with RSV cases.

**Table 2:** Correlation of meteorological factors with RSV cases

Meteorological factors	Range	Mean ± standard deviation	Spearman correlation coefficient	p	Multiple regression coefficient (B)	p
Temperature (°C)	25.7-29.6	27.4 ± 0.7	-0.116	0.037*	-2.426	< 0.001**
Relative humidity (%)	71.6-86.8	79.7 ± 3.0	-0.082	0.141	-1.070	< 0.001**
Rain days (n)	4.5-28.5	16.9 ± 4.7	0.147	0.008*	0.469	< 0.001**
Pressure (hPa)	1007.1-1013.2	1009.8 ± 0.9	0.095	0.089	NS	
Rainfall (mm)	10.4-585.9	235.9 ± 112.2	0.102	0.067	NS	
UV radiance (Mjm^-2^)	11.2-20.1	15.9 ± 1.5	0.010	0.857	NS	

NS, Not significant

* p < 0.05.

** The combined R^2 ^for temperature, relative humidity, and rain days was 0.143.

## Discussion

The Malaysian Ministry of Health reports that respiratory infections are one of the principal causes of hospitalization (9.4% in 2009), and pneumonia is one of the ten principal causes of death (10.4% in 2009) in public hospitals [10]. However, as diagnostic capacity for respiratory viruses is extremely limited, little is known about the epidemiology of viral RTIs in Malaysia, which are well known to have high financial and clinical impact [11-13]. This retrospective study of respiratory viruses at a teaching hospital over the past 27 years is the most comprehensive study carried out in Malaysia. Our findings support a previous local study carried out over a year, which showed that RSV was the most commonly detected respiratory virus, followed by parainfluenza viruses, influenza viruses and adenovirus [7]. We found that this trend has remained for the past three decades.

In our laboratory, the average virus detection rate (annually) by IF and/or virus isolation was 25.4% in all respiratory samples tested. This considerably underestimates the true burden of respiratory viruses, as lack of resources precluded the routine use of more sensitive diagnostic methods such as shell vial culture [14] and molecular detection methods, and testing of a wider range of viruses. Respiratory virus detection rates of more than 50% can be achieved with molecular detection [15,16]. Other studies in tropical countries have reported that emerging respiratory viruses such as metapneumovirus (5.3-5.4%) [17,18], coronaviruses (0.6%) [19], bocavirus (8.0%) [19] and human rhinovirus C (12.8-30%) [20,21] also contribute substantially to morbidity.

Most studies, including those in Asia, show that the most common causes of respiratory viral infections are RSV and rhinoviruses [3,22-24]. Our laboratory does not routinely detect rhinoviruses, but RSV was the most frequently detected respiratory virus, particularly in infants less than one year old. This suggests that maternal antibodies were ineffective in preventing RSV infections. Older children may be less prone to RSV infection due to the maturity of their immune system or natural immunity obtained through repeated infections of RSV. The burden of RSV in young infants in tropical countries, including Malaysia, emphasizes the likely global benefits of developing a safe and effective vaccine for RSV. Our data also showed a slight male preponderance (59.8%) in children with respiratory viral infections, consistent with other studies [25].

In temperate countries, respiratory viral infections have clear seasonal variations with most cases occurring during winter. Possible explanations for this include seasonal variations in host immune response to infection [26], climatic factors such as ambient temperature and low relative humidity which increase viral survival in the environment [27], and changes in host behaviour which increase contact with others. Seasonal trends are more variable in the tropics, with some studies showing that respiratory virus infections occur all year round, while some show clearer seasonality.

Located in Southeast Asia, Kuala Lumpur (latitude 3°N) has a mean annual temperature of 27.4°C, constant high relative humidity (> 71.6%), and heavy rainfall throughout the year. In our study, the common respiratory viruses were detected throughout the year. The RSV annual epidemics are strongly seasonal, while seasonality is less clear for influenza, parainfluenza, and adenovirus, which may be due to the smaller number of cases. For adenovirus, most studies show sporadic occurrence without any seasonal trend [28,29]. In the tropics, influenza occurs all year-round [29], similar to our data. In Singapore, parainfluenza virus type 3 was the most commonly detected parainfluenza virus type, with seasonal peaks in February-March similar to our results [29].

In Kuala Lumpur, RSV peaks at the end of the year. In contrast, in neighbouring Singapore (latitude 1°N) and Lombok, Indonesia (latitude 8°S), RSV peaks around March-August [29,30]. In most RSV studies carried out in temperate countries, where temperature varies widely between seasons, temperature is highly inversely correlated to RSV cases [4,31]. We also found a negative correlation between RSV and temperature in tropical Kuala Lumpur, where the much reduced temperature variation may explain the weaker correlation (correlation coefficient of -0.116). As previously observed in Indonesia [32], we also found that the number of rain days was significantly associated with RSV cases, but not rainfall. Malaysia experiences brief, intense showers of rainfall, as well as prolonged episodes of light rainfall. Therefore, the number of rain days may have a greater influence than the absolute amount of rainfall on behaviours such as children staying indoors, thus increasing close contact and indoor transmission of respiratory viruses [33].

While humidity was inversely correlated with RSV in our study, as with respiratory infections in Singapore [5], a positive correlation was reported in Lombok, Indonesia [32]. There are well recognized inconsistencies in reported associations of respiratory infections with meteorological factors in different settings [34]. Clearly, seasonality of respiratory viruses in the tropics cannot be explained by climatic factors alone, as associations vary widely between geographic locations [35]. There are likely to be multiple, poorly understood interactions between climatic, environmental and behavioural factors, and complex interplay between different circulating viruses and population immunity. The local epidemiology of respiratory viruses needs to be determined for each site, for effective planning of interventions such as potential vaccines.

This study has several limitations. As the data was collected retrospectively, there was some missing data. We were unable to obtain dates of admission for the earlier patients, and thus could not differentiate between nosocomial and community-acquired viral infections. However, we have previously found that nosocomial cases made up 25/157 (15.9%) influenza cases from 2002-2007 [13] and 17/146 (11.6%) RSV cases from 1989-2010 (Khor CS, Sam IC and Chan YF, unpublished observations). These nosocomial rates of respiratory viral infections are similar to previously published rates of 12.1-13.8% [36,37], thus supporting the likelihood that the majority of infections seen in our study were community-acquired. Over 27 years, there may have been changes in physicians' practices, such as criteria for taking specimens and admitting patients, and types of samples collected. Furthermore, with a relatively low IF sensitivity of 55.8% using virus isolation as the gold standard, there is likely to be considerable underdiagnosis of viral infections compared to molecular methods [38]. Nevertheless, despite these limitations, these unusually extensive records kept over 27 years provide valuable insight into current and historical respiratory virus epidemiology in a tropical Southeast Asian country, particularly with the limited available facilities for virus diagnosis. To extend these findings, we are currently carrying out prospective studies using molecular methods to detect a wider range of respiratory viruses.

## Conclusion

Respiratory viral infections due to RSV, parainfluenza viruses, influenza viruses and adenovirus are significant causes of morbidity in hospitalized children in Kuala Lumpur, and are likely to be underdiagnosed. The most common cause of viral RTI was RSV, which causes annual seasonal epidemics and predominantly infects young infants ≤ 6 months. Further studies, both hospital-based and population-based, are required in Malaysia to fully understand the clinical and community impact of respiratory viruses in children.

## Methods

### Study population

University Malaya Medical Centre (UMMC) is a 900-bed tertiary hospital in Kuala Lumpur, the capital of Malaysia. In this retrospective study, we analysed records of all respiratory samples from hospitalized children aged ≤ 5 years sent to the hospital's diagnostic virology laboratory between 1982-2008. The decision to admit children and take respiratory samples was made at the discretion of the attending physician. Most (> 90%) of the respiratory samples received were nasopharyngeal secretions or aspirates, with other specimen types including bronchoalveolar lavages, oropharyngeal secretions, nasal swabs, tracheal secretions, throat swabs, and sputum. Duplicate positive samples collected from the same patient within a week were removed from analysis. As all other samples received were analysed, regardless of the timing during admission, nosocomial infections cannot be excluded. Co-infections were counted as separate cases. Ethics approval was obtained from the Medical Ethics Committee of University Malaya Medical Centre (reference number: 788.3).

### Laboratory procedures

All respiratory samples were routinely screened for respiratory viruses by direct IF and viral isolation. Cells obtained by centrifuging clinical samples were fixed onto slides, and IF for the common respiratory viruses (influenza A and B, parainfluenza 1, 2, and 3, RSV, and adenovirus) was performed using Light Diagnostic Respiratory Panel 1 Viral Screening & Identification Kit (Millipore, Billerica, USA) according to the manufacturer's instructions. Viral isolation was performed by inoculating the respiratory samples into Madin Darby canine kidney (MDCK; ATCC number CCL-34), Vero (ATCC number CCL-81), A549 (ATCC number CCL-185), and HEp-2 (ATCC number CCL-23) cells, and incubated at 32°C with 5% CO_2_. Infected cells were monitored daily for up to 10 days, and those showing cytopathic effect (CPE) were harvested for IF. For samples with no CPE after ten days, haemadsorption with human type O blood was performed on MDCK cells to detect influenza viruses. A laboratory-confirmed or positive case of respiratory virus infection was defined as a case with positive IF and/or virus isolation.

### Statistical analysis

Analysis was carried out with SPSS v16.0 (SPSS Inc., Chicago, USA). Demographic factors such as gender, age, and ethnicity were analyzed by univariate ANOVA. Levene's test, Brown-Forsythe test and Welch test were done to confirm the homogeneity of variance across samples. Since the data variance is unequal, the Games-Howell test was selected as the post hoc test to compare cases with each virus type to the population separately. The seasonal trends for each respiratory virus were evaluated.

As the number of RSV cases was highest, RSV activity was further analyzed to determine its associations with meteorological factors such as rain days, relative humidity, temperature, pressure, rainfall, and ultraviolet radiance. Meteorological data were provided by the Malaysian Meteorological Department, from two weather stations located in Petaling Jaya and Subang Jaya, about 5-15 km away from the hospital. The monthly data from both stations were averaged before correlation with monthly RSV cases. Spearman's rank correlation and stepwise multiple regression analysis and were performed with RSV cases as the dependant variable, and the climatic factors as independent variables. A p value of < 0.05 was considered significant. The smaller numbers of cases precluded similar analyses for the other respiratory viruses.

### Reviewers' comments

#### Reviewer

Hannah Moore

1. The methods section needs to follow the background section and not be placed at the end of the manuscript.

#### Authors' response

We have submitted the manuscript in accordance with the BMC Pediatrics instructions for authors, which asks for the methods section to be placed after the conclusions section (http://www.biomedcentral.com/bmcpediatr/authors/instructions/researcharticle).

2. Table 1 column headings can still be improved. Column headers should include "n(%)"

#### Authors' response

Included in the table.

3. I believe it would be useful to display data < 6 mths and 6-12 mths, however I will leave that decision to the Associate Editor

#### Authors' response

We agree that the age group data would make the paper more useful. The data for < 6 mths and 6-12 mths have been included in Table 1.

4. Figure 3 is useful but is difficult to see in grayscale. It needs to be produced in colour or different shading patterns are required. It appears that adenovirus testing did not occur until 1991. If that is so, this needs to be described in results.

#### Authors' response

We have provided Figure 3 in colour. Adenovirus testing has been performed since 1982, and in fact Figure 3 does show that adenovirus was detected in very low numbers in 1985, 1987 and 1990. We have modified the colour of the adenovirus bars to make it clearer.

5. Results text describing the increase of number of samples over the years and the positive detection rate remaining stable at 25.4 +/- 8.1%, needs to be described more clearly. Presumably this is overall rate and standard deviation? A range of the virus positivity rate between the years would be more useful.

#### Authors' response

We have clarified that 8.1% is the standard deviation, and added the range to the sentence, as suggested. We hope that it is now clearer.

"Quality of written English: Needs some language corrections before being published"

#### Authors' response

The manuscript has been reviewed and corrected by the two main authors who conceived the study (ICS and YFC), who are both native English speakers. Furthermore, ICS received his secondary, university and postgraduate education in English in the United Kingdom.

##### Reviewers

This article was reviewed by Hong Yan Zhang and Hannah Moore (both nominated by Associate Editor Dat Tran).

##### Open peer review

Reviewed by Hong Yan Zhang and Hannah Moore (both nominated by Associate Editor Dat Tran). For the full reviews, please go to the Reviewers' comments section.

## Competing interests

The authors declare that they have no competing interests.

## Authors' contributions

The study was conceived by YFC and ICS, who supervised CSK in study design and data collation. PSH collected the original diagnostic laboratory data. KFQ planned the statistical analysis. All authors were involved in analysis of data and writing of the manuscript. All authors have read and approved the final manuscript.

## Pre-publication history

The pre-publication history for this paper can be accessed here:

http://www.biomedcentral.com/1471-2431/12/32/prepub
